# Influence of the orientation of constructed blood vessels during the 3D printing on the measurement of the pseudo-oxygen saturation of an artificial blood substitute using conventional oxygen sensors: a test series

**DOI:** 10.1186/s41205-024-00246-7

**Published:** 2024-11-27

**Authors:** Svenja Jung, Martin Hoffmann, Dirk Winkler, Erdem Güresir, Fabian Kropla, Sebastian Scholz, Ronny Grunert

**Affiliations:** 1 Department of Neurosurgery, University of Leipzig Medical Center, Liebigstr.20, 04103 Leipzig, Germany; 2 Fraunhofer-Institute for Machine Tools and Forming Technology, 02763 Zittau, Germany

**Keywords:** 3D print, 3D printed vessel, Additive manufacturing, Phantom, Vascular phantom, Oxygen sensor, Blood substitute, R-value

## Abstract

**Background:**

The development of phantoms to reduce animal testing or to validate new instruments or operation techniques is of increasing importance. For this reason, a blood circulation phantom was developed to test a newly designed retractor system with an integrated oxygen sensor. This phantom was used to evaluate the impact of the 3D printed blood vessel on the measurement of the oxygen saturation.

**Methods:**

A solution of nickel sulfate and copper sulfate was prepared as a substitute for real blood. The absorption spectra of these solutions were recorded and compared with those of blood. Subsequently, the oxygen sensor used was calibrated to the blood substitute. Additionally, blood vessels with a simplified geometry were designed and manufactured using inverted vat polymerization and an elastic material (*Formlabs Elastic 50 A*). To determine the orientation during the printing process, various vessels were printed. Measurements to assess the effects of disturbance (rotation of the vessels during measurements) on the sensor readouts were prepared.

**Results:**

The impact of disturbances was verified through the rotation of the 3D printed vessels. It was demonstrated that a direct measurement on the disturbances led to outliers and higher values. An optimal orientation was determined to be a lateral placement (90° or 270°) of the sensor. Regarding the orientation of the vessels within the printing space, an orientation of 45° yielded the best results, as the individual layers had the least impact on the light emitted and received by the oxygen sensor.

**Conclusion:**

The achieved results demonstrate the influence of the orientation of the vessel during 3D printing as well as the influence of the position of the vessel during the measurement using a conventional oxygen sensor.

## Background

Across Germany, over five million animals were used for research and development of various medical and cosmetic products in 2021. More than half of these animals (> three million) were subsequently killed during or after testing. This puts Germany in first place in terms of animal usage in Europe, above France and Norway [1, 2].

To reduce the numbers, the development and use of artificial phantoms is a step in the right direction. Therefore, the developed simulation models can be used to validate new types of tools or to learn surgical techniques. These consist of thoracic- and organ phantoms [3–8], tissue phantoms [9–12] and head and brain phantoms [13–19], which are especially significant for neurosurgery. This also includes neurovascular phantoms that reflect the anatomy of the vessels inside the brain. In 2019, a research group from Hamburg developed the simulation model ‘HANNES’ to learn, for example, the skills for treating intracranial aneurysms [20]. The corresponding blood vessels were produced by using 3D printing. In this study, a mixture of water and soap or glycerol was employed as a blood-like medium [21]. However, this mixture lacks the optical properties of blood and is therefore unsuitable for use in phantoms that require oxygen sensors. For such applications, hemoglobin, typically extracted from bovine blood, is commonly used (e.g [22, 23]). An alternative approach is to utilize materials such as dyes [24] or copper and nickel sulfate [25, 26] can be used to produce exclusively artificial phantoms.

The aim of this study was the development of a vascular phantom to test a newly designed retractor system to measure cerebral oxygen saturation during surgery. An oxygen sensor was integrated into the tip of the retractor. In order to create a suitable phantom, it was necessary to determine the impact of the material on the sensor’s oxygen measurements and to evaluate whether adjustments to the blood substitute or recalibration of the sensor would be required.

For that reason, the objective of this investigation was to determine the impact of 3D printing on the optical properties of a blood substitute. For this purpose, an artificial blood circulation system with a 3D printed blood vessel was developed, allowing the effect to be evaluated using a conventional oxygen saturation sensor.

## Methods

### Experimental setups

**Fig. 1:**
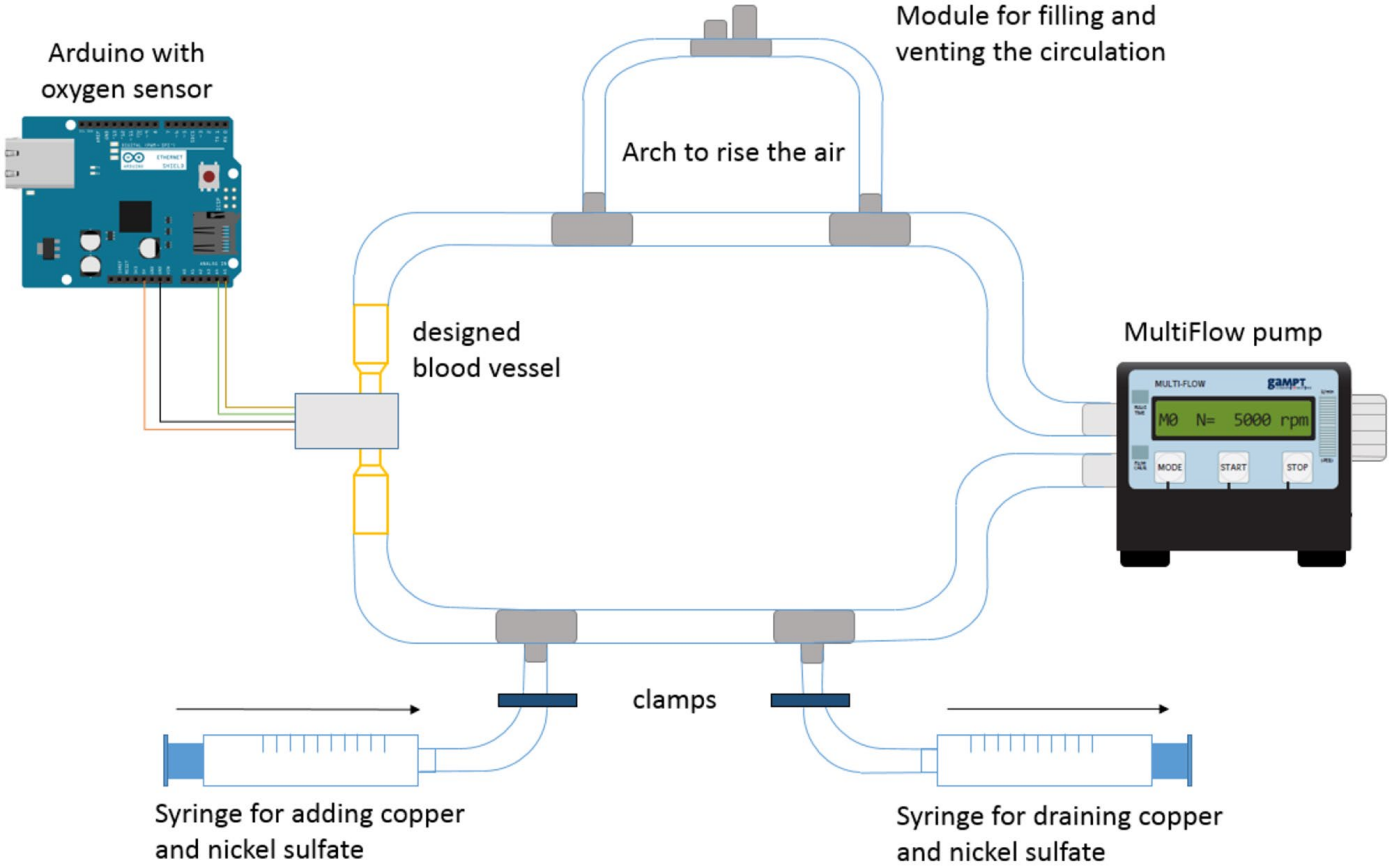
Schematic representation of the simulated blood circulation with the components

The developed test setup for determining the influence of printed blood vessel orientation on the measurement of pseudo saturation consists of an oxygen sensor (*Max30102)* by *Whadda (Gavere*,*Belgium*), an Arduino (*Arduino UNO Rev3 DIL Core ATMega328) by Conrad Electronic SE (Hirschau*,*Germany)*, a *MultiFlow-pump* by *GAMPT (Merseburg*,*Germany)*, multiple hoses by *Elmar Jung Product Solutions GmbH & Co. KG (Neunkirchen*,*Germany)* and *Hubert & Piening Handels GmbH (Harpstedt*,*Germany)*, 3D printed connectors, and a 3D printed blood vessel (Fig. 1). A substitute of copper and nickel sulfate is used as the medium to simulate the blood. To ensure an air-free system, an arch was integrated to allow the rise and accumulation of air. Two syringes by *Romed (Wilnis*,*Netherlands)* were connected for adding or removing fluid.

### Copper and nickel sulfate as the medium for the blood substitute

A solution of copper sulfate (*copper(II)-sulfate pentahydrate by Carl Roth GmbH + Co. KG; Karlsruhe*,*Germany)* and nickel sulfate (*nickel(II)-sulfate hexahydrate by Carl Roth GmbH + Co. KG; Karlsruhe*,*Germany)* was chosen as medium for blood simulation. To create different solutions with different simulated oxygen saturations, a 2.2 molar nickel sulfate (NiSO_4_) and a 0.5 molar copper sulfate (CuSO_4_) solution were used. The pseudo-saturation indicates how much %Vol of copper sulfate is present in the solution. For example, a solution with a pseudo-saturation of 70% contains 70%Vol of copper sulfate and 30%Vol of nickel sulfate.

**Fig. 2:**
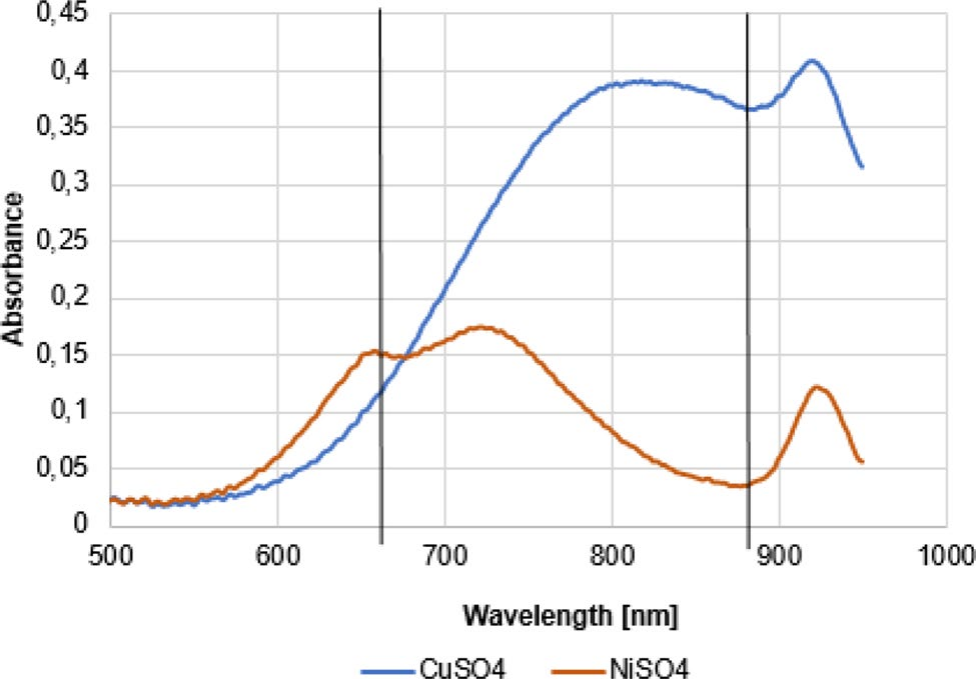
The absorption spectrum of the synthesized blood substitute

To compare the blood substitute with the absorption characteristics of real blood, the absorption spectrum of the two substitute solutions was recorded using the *Novaspec Plus spectrophotometer* from *Amersham Bioscience (Slough*,*UK)* (Fig. 2).

**Fig. 3:**
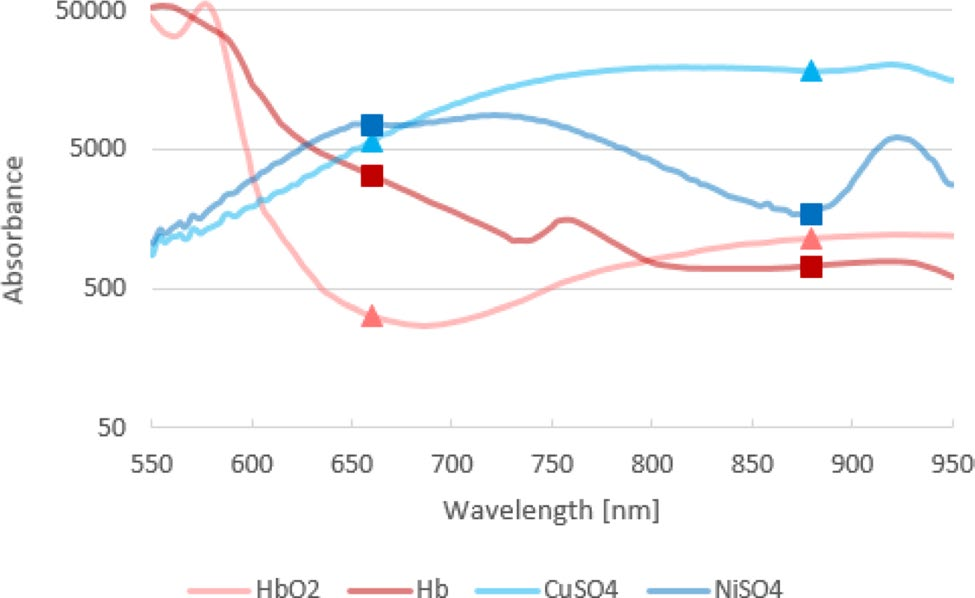
Comparison between blood substitute and blood components – visualization of the relevant wavelengths (660 nm and 880 nm)

The recorded absorption values of real blood were taken from Scott Prahl [27]. For better visualization and thereby a better comparison of the two recorded absorption spectra, the absorption values for the blood substitute were increased by a factor of 50,000 (Fig. 3). According to the Beer-Lambert law, this did not affect the trend of the individual curves.

The absorption values at 660 nm and 879.6 nm are marked for comparison. These wavelengths are detected by the oxygen sensor used and are included in the calculation of the R-value. The R-value is the ratio of the absorbance amplitudes of the applied medium under red (660 nm) and infrared (879.6 nm) light [28]. This numerical value provides information about the existing oxygen saturation.

**Fig. 4:**
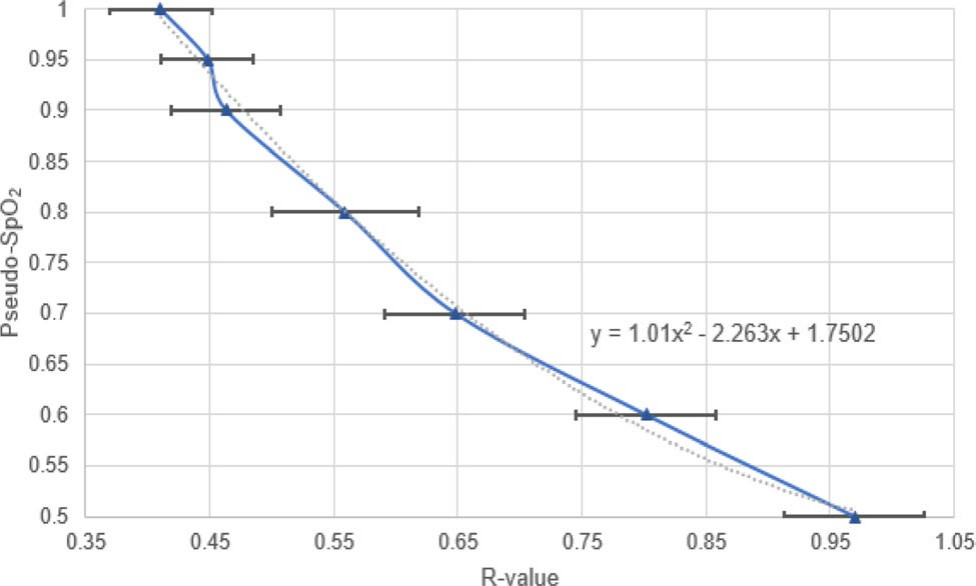
Representation of the R-value across the pseudo-saturation of the blood substitute for sensor calibration

Figure 3 shows that the hemoglobin (Hb) and oxyhemoglobin (HbO2) curves of blood (red) are comparable to the absorption curves of the blood substitute (blue). Copper sulfate mimics oxyhemoglobin and nickel sulfate mimics hemoglobin. To calibrate the oxygen sensor for the used blood substitute, the R-values of various solutions with a pseudo-saturation of 50–100% were determined by using 15 absorption spectra each, and the corresponding calibration curve was calculated (Fig. 4).

Unlike conventional pulse oximeters, which have an idealized linear calibration curve for the 70–100% range, we chose a quadratic regression for measurement accuracy and range.

### Selection of a suitable 3D printing process for vascular phantoms

Because of the physical requirements for printing complex, hollow structures such as the neurovascular tree, 3D printing solutions that best emulate human anatomy use material jetting. The most studied example is the neurovascular system where very complex and high risk procedures can be simulated both in the digital space and by using 3D printed parts [29–34]. Additional work includes studies conducted by Kaschwich, Horn et al. [35] and Dorweiler, Baqué et al. [36].

Less expensive alternatives 3D printing technologies can be used. While these have drawbacks (e.g. ensuring the lumen is smooth and uniform) with respect to specific patient care opportunities, they can prove sufficient for testing strategies such as those in this project. For example, artificial blood vessels can be 3D printed using inverted vat polymerization (to include stereolithography (SLA) as one example), material extrusion (to include fused deposition modelling (FDM) for example), and powder bed fusion (to include SLS, SLM). Printable materials include metals and hard plastics, such as polylactic acid (PLA), as well as flexible materials, such as thermoplastic polyurethane (TPU) [36–38].

In this study, the criteria for the physical parts included using a transparent and flexible material to securely affix the light sensor and accurately replicate a non-rigid blood vessel. Powder bed fusion was not a viable option due to the utilization of opaque materials, which did not align with the transparency requirement. The decision to utilize stereolithography was ultimately made on the basis that, at the time of the study, neither a material jetting nor a material extrusion printer with the requisite transparent and flexible materials was available.

### 3D printed blood vessels

**Fig. 5:**
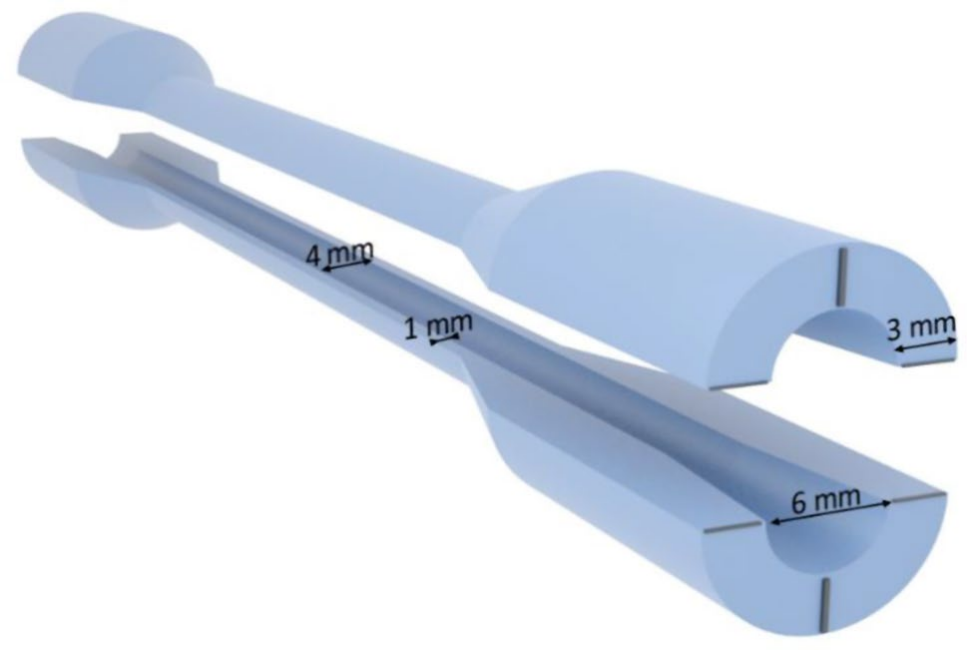
Illustration of the constructed blood vessel with the corresponding dimensions

As a consequence of the inherent limitations of the 3D printing technology employed (stereolithography), the shape of the vessel models was greatly simplified and coils and twists were removed. A hollow cylinder with an inner diameter of 4 mm and an outer diameter of 6 mm was designed. A larger wall thickness (3 mm) was used for the ends of the cylinder to prevent material tearing when attaching adapters for the circulation (Fig. 5).

The vessels were 3D printed using the *Form 2* printer from *Formlabs (Sommerville*,*USA)*. The material that was used was *Resin Elastic 50 A from Formlabs*. Support structures were attached to enable the 3D printing. After printing, the vessel models were placed in a 99.9% isopropanol bath (*Form Wash*, *Formlabs*) for 20 min to remove any residual liquid resin. The vessel models were then cured using the *Form Cure* curing station (*Formlabs*) for 60 min at 60 °C under UV light.

**Fig. 6:**
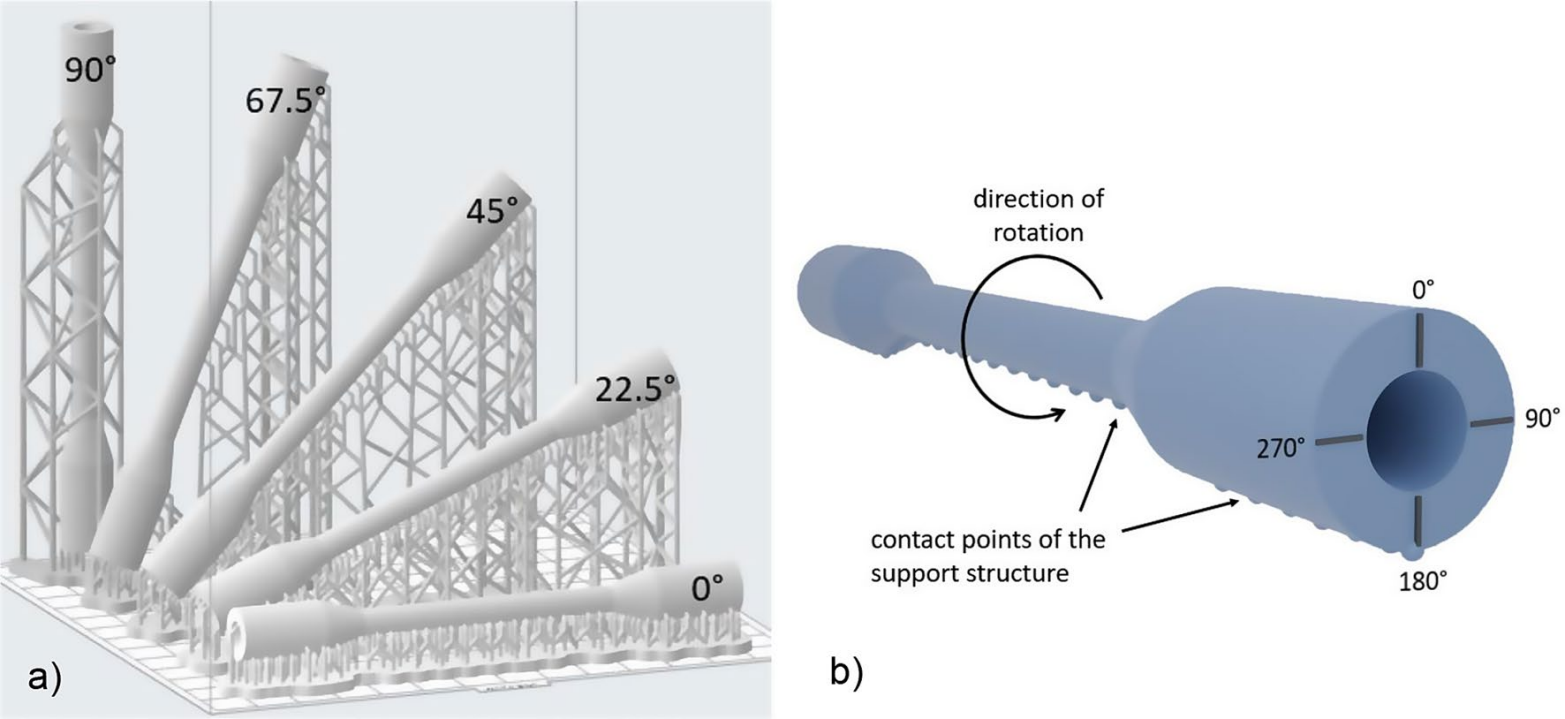
Illustration of the two mentioned factors of influence and their realization in the measurement. **a**)Presentation of the various orientations of the constructed vessels in the printing space **b**) Presentation of the rotation points of the constructed blood vessels by using the oxygen sensor

Once the support structures had been removed, the contact points and the individual print layers were clearly visible. For this reason, in addition to the influence of the applied material, the effect of artifacts on the R-values measured by the oxygen sensor was examined. To investigate the influence of the layer orientation within the models, the vessel model was printed in five different orientations relative to the build platform. The defined orientations were 0° (horizontal to the platform), 22.5°, 45°, 67.5° and 90° (vertical to the platform) (Fig. 6a).

After printing, the different vessel models were sequentially introduced into the circulation and the measured values recorded. To fully evaluate the influences of the layer orientation as well as the influences of the contact points of the support structure, the individual vessel models were rotated at defined 90° angles along the long axis of the vessel. There are no contact points at 0°, only a few at 90° and 270°, and many at 180° that can impact the sensor’s light transmission (Fig. 6b).

### Results

During printing of the designed vessels, it was found that the printing of a 90°-aligned vessel was not possible, resulting in tearing and destruction of the vessel. Accordingly, no values could be gathered for this. Subsequent measurements were conducted using a blood substitute solution, i.e., the concentration of the solution remained constant throughout the measurements.

**Fig. 7:**
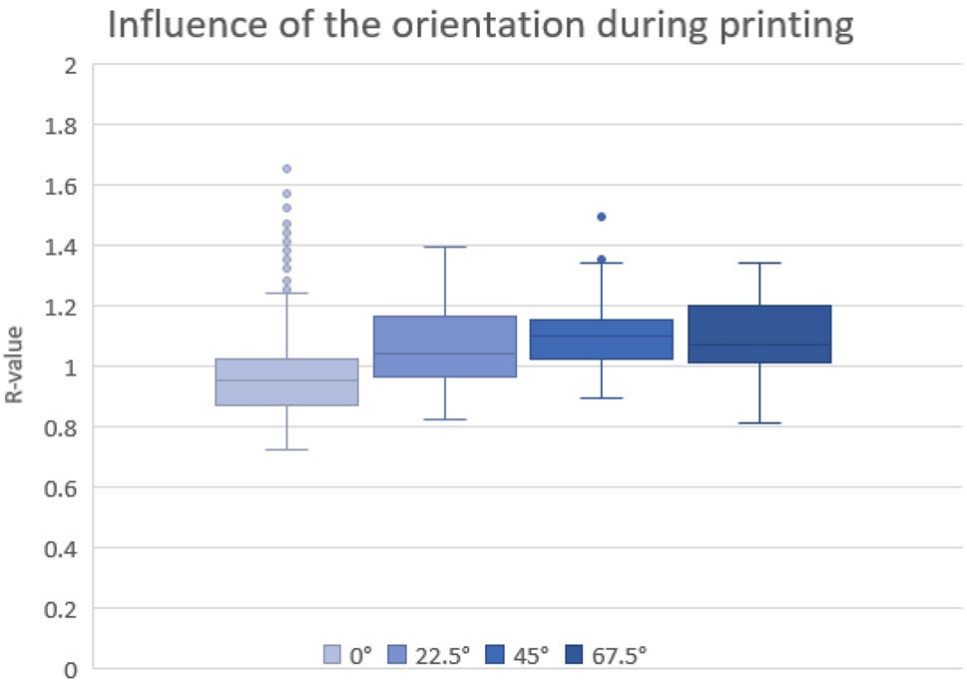
Illustration of the influence of the orientation of the constructed blood vessels during printing

Figure 7 illustrates the results for evaluating the orientation of the designed vessels in the print space. It is evident that the blood vessel with an orientation of 0° exhibits the lowest average values. This is followed by the vessels with a positioning of 22.5° and 67.5°. The vessel with an orientation of 45° had the highest median value. It should be noted that the dispersion for this orientation, except for 0°, was the lowest compared to the others (see box size). Furthermore, the chart clearly indicates that there are many outliers at 0°, whereas there are few to none in the other orientations.

**Fig. 8:**
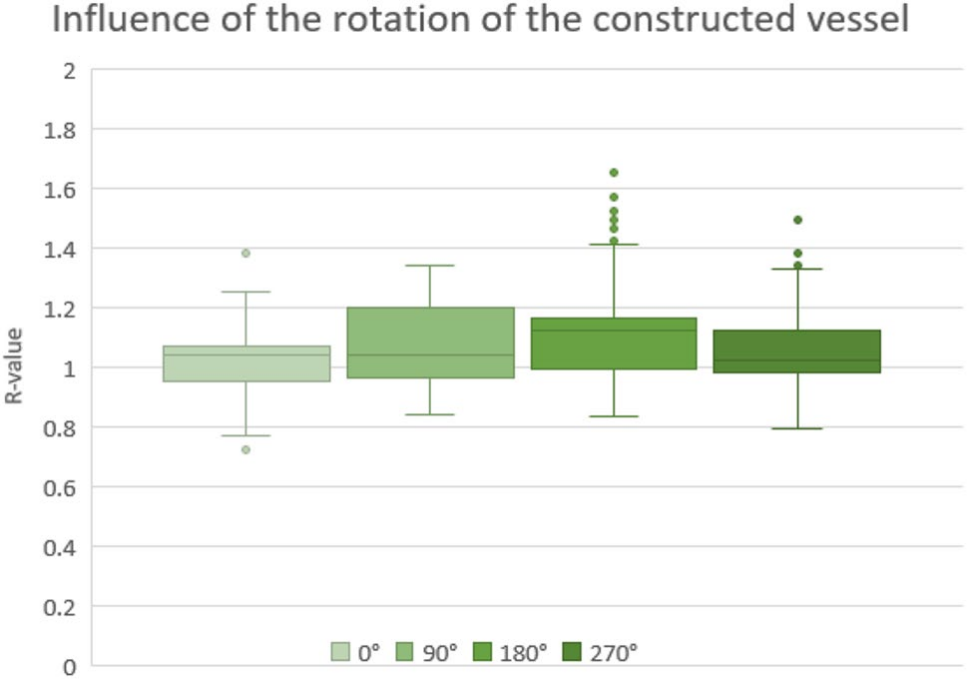
Illustration of the rotational impact of the constructed blood vessels on the measurement values

Figure 8 shows the results of rotating the constructed blood vessels. The average values are close to each other, but at 180°, corresponding to the contact points of the support structure, the values are higher. Additionally, all recorded values for the different rotations exhibit fluctuations (larger boxes, larger whiskers), and outliers (except at a 90° angle).

## Discussion

### 3D printing of the designed vessels

**Fig. 9:**
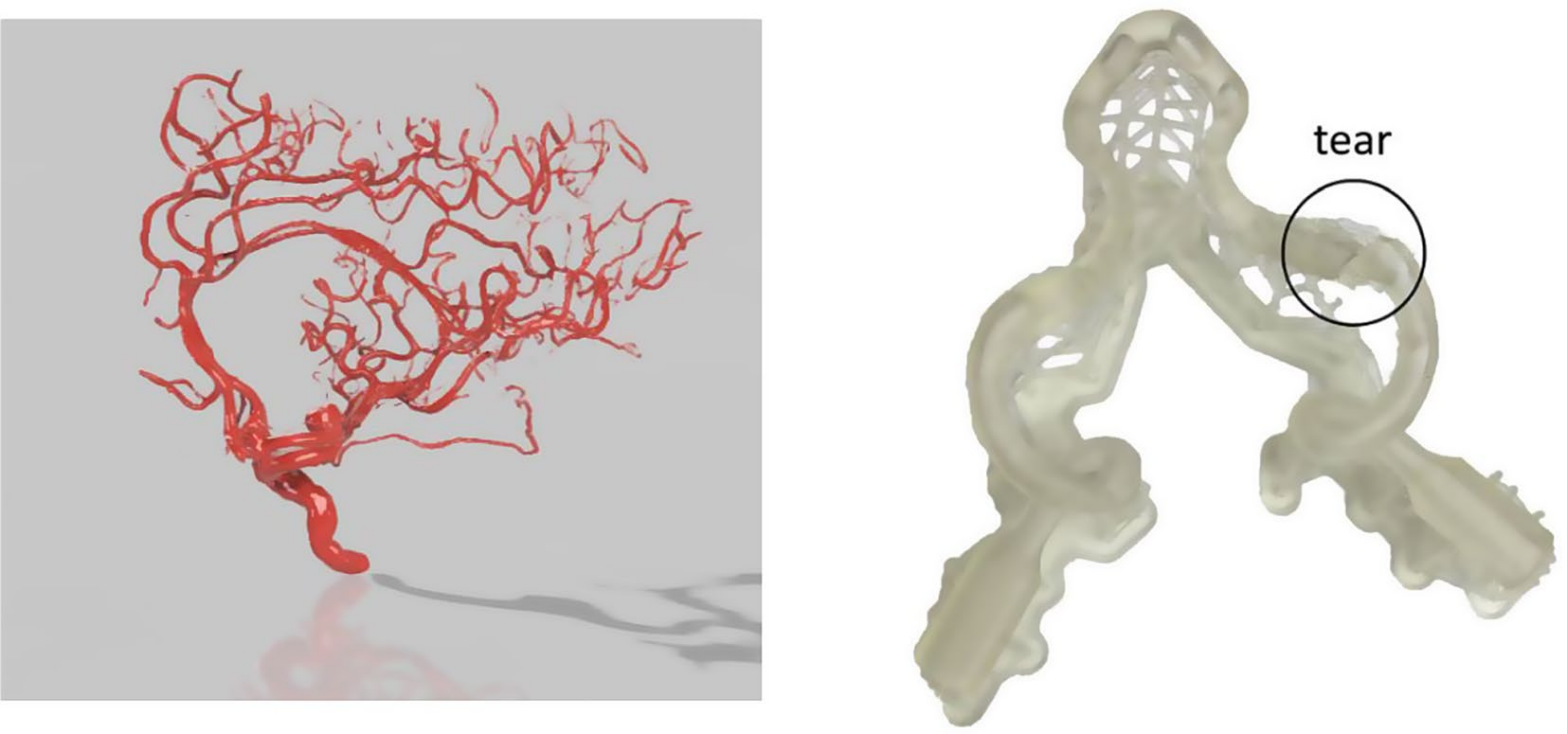
A 3D model of a hollow vascular tree(left) and an Illustration of a rupture in a more complex vascular model (right)

The Form 2 was utilized to create the vessel model. The design of the vessel was significantly simplified for this study due to the unsuccessful printing of more complex structures, including hollow vascular trees and replicas of twisted vessels (Fig. 9).

One limitation of this study is that the complexity of the human vascular system could not be replicated, based on the use of desktop inverted vat polymerization for 3D printing. However, the data derived from this study can be translated to more sophisticated 3D printing and simulation of important procedures [29–34].

Regarding other methods to 3D print transparent parts, filaments for material extrusion printers are available in transparent forms. However, the currently available transparent and flexible materials exhibit a Shore hardness that is predominantly higher than that of the resin used. As a consequence of developments in the field of support structures, it is now feasible to print water-soluble supports made from, for example, polyvinyl alcohol (PVA) using this method. This allows the creation of intricate structures, such as a vascular tree, and their removal without causing defects. These and other technologies (e.g., material jetting), as well as flexible materials such as TPU, ABS (acrylnitrile butadiene styrene), and silicones, will be the subject of future studies.

### Orientation of the designed vessels during 3D printing

In consideration of the 3D printed vessels, it can be identified that all but the one with a 90° orientation to the build platform could be utilized. In order to ensure successful 3D printing with an elastic material, care should be taken to align the largest surface of the object to be printed parallel to the build platform. Therefore, elongated objects, such as simplified blood vessels, should be positioned as flat as possible to maximize the base area and thus the raft on which the support structures are anchored. Since the raft is minimal when the blood vessel is oriented at 90° to the printing platform, this prevents successful printing.

### Influence of the orientation of the designed vessel during the 3D printing

**Fig. 10:**
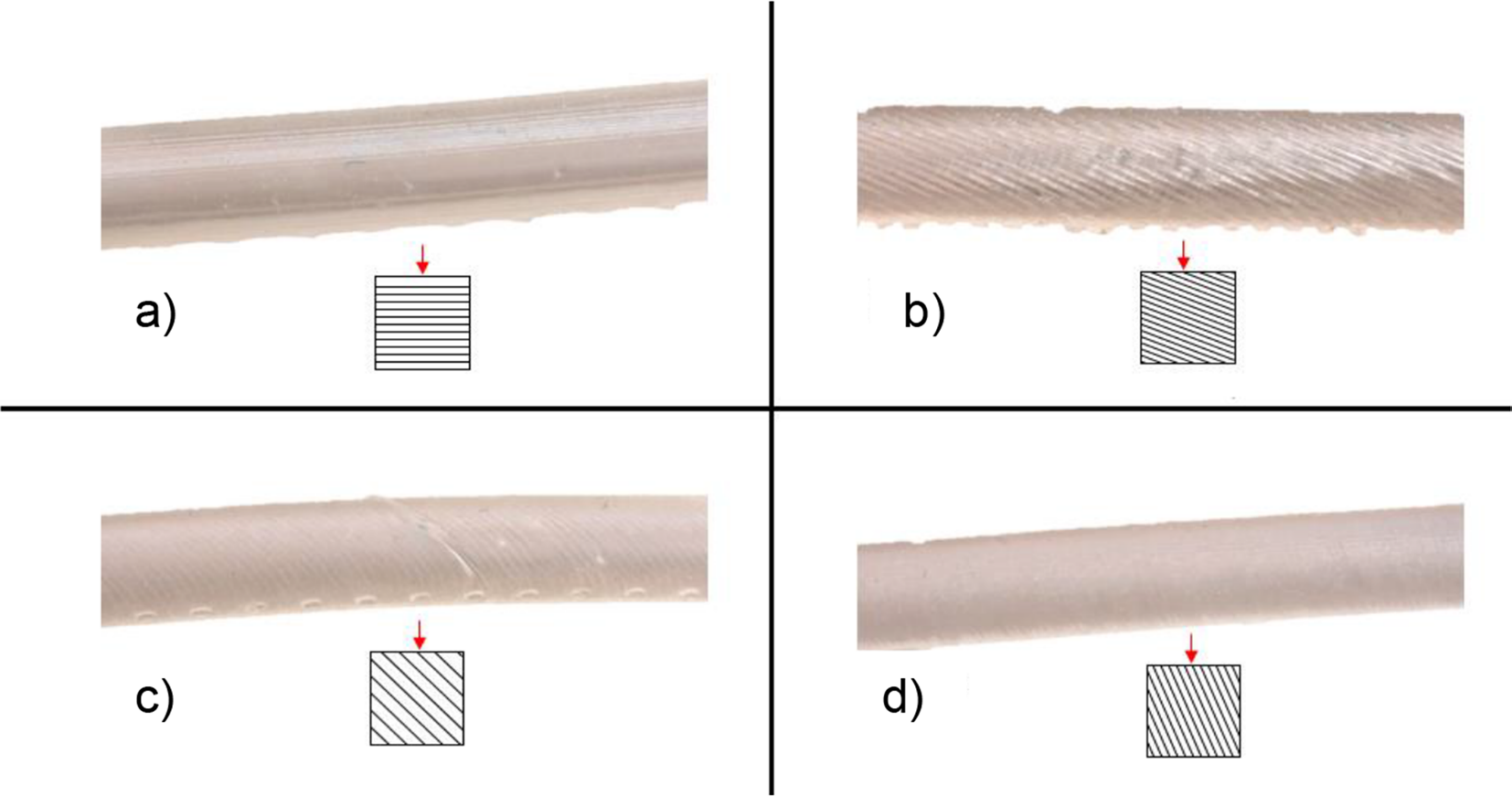
Representation of layer boundaries at different orientations in the print chamber. **a**) 0° orientation -, **b**) 22.5° orientation -, **c**) 45° orientation - and **d**) 67.5° orientation of the constructed vessel

Due to the layer-by-layer application of the resin, the orientation of the objects during printing has an impact on the measurement values. Figure 10 shows the layer directions for each print orientation.

The orientation of the printed layers relative to the sensor can result in varying refraction and reflection phenomena. Since the sensor remains stationary and is consistently applied to the blood vessels in a fixed manner, the sensor is only capable of detecting the effects of the printed layers.

In general, Fig. 7 shows that the mean of each measurement increases slightly with increasing orientation. At an orientation of 67.5°, there is a slight drop, but the scatter is significantly larger. Technically, the best method for printing the vessel is an orientation of 0°, since this maximizes the surface area perpendicular to the build platform. However, the post-processing of the vessel is more extensive, as resin accumulates inside the vessel during printing and must be removed. Furthermore, significant outliers occurred. These can arise due to the quality of the print or improper alignment of the sensor, leading to increased reflections at the individual layers. Additionally, cracks occurred along the layer boundaries during the measurements, causing the destruction of the vessel.

Based on post-processing and print stability, a 45° orientation is the best method for creating a simplified blood vessel. This method allows the resin to flow out during printing, while ensuring that the necessary support structure remains at an optimal length to minimize the risk of breakage and printing errors.

### Influence of the rotation of the designed vessel during the measurements

**Fig. 11:**
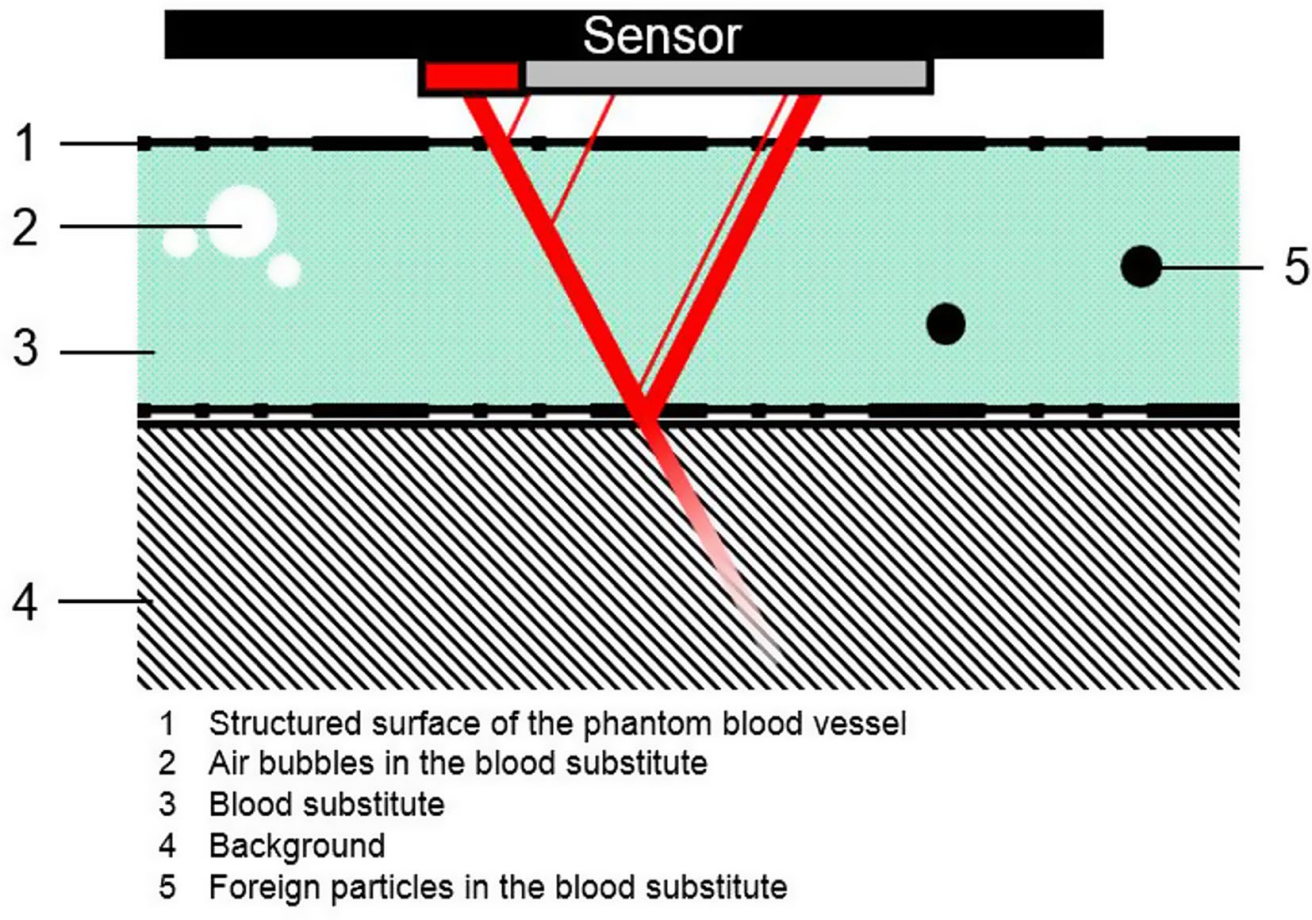
Schematic representation of the sensor’s beam path and illustration of sources of interference

Figure 8 illustrates that the sensor positioning at an angle of 180° is not optimal due to the presence of support structures. Although small burrs may remain after removal, and so the reflective properties of the light at these contact points differ from those at other areas of the constructed vessel. Consequently, during the use of a sensor, unintended changes of the optical properties (e.g. reflection) at the surface can negatively influence the measurement and cause fluctuations or outliers. Due to these properties, positioning at 0° should also be neglected. This position is perpendicular to the support structures, leading to an influence of their contact points since, as the wall thickness varies, affecting the optical properties of the emitted light (Fig. 11).

In summary, using a stereolithographic printer, the following statements can be made regarding the optical properties: The printing orientation should be chosen so that the length of the support structures is minimal and the liquid resin can flow out of the hollow structure. As far as optical properties are concerned, the orientation of each layer affects the possible refractions and reflections. Therefore, it must be chosen according to the sensor used. In the case presented here, the chosen orientation would be 45°, excluding outliers. To position the sensor on the printed object, it is recommended to place it laterally to the support structures. This ensures that the points of the support structure are not in the beam path of the sensor and therefore do not affect the resulting values.

### Conclusion

The development of artificial phantoms to represent various medical conditions, as well as to support teaching and testing of novel surgical tools, has gained increasing recognition in recent years. In order to develop a phantom with integrated blood circulation for testing a newly designed retractor, a system was created that incorporates an artificial, 3D printed, transparent and flexible blood vessel, along with a blood substitute which is capable of measurable oxygen saturation. The efficiency of 3D printing orientation and rotation due to the oxygen sensor was evaluated in relation to the optical properties of the blood substitute. For the blood vessel, a simplified shape was designed and printed using stereolithography with an elastic material (*Elastic 50 A* from *Formlabs*).

Since the impact of the material on the oxygen saturation measurement was unknown at this time, two factors of manufacturing and measurement setup were investigated. The initial step involved a detailed examination of the five different orientations of the vessels during the printing process. Secondly, the influence of the support structure on the sensor was considered by examining the vessel at four different angles. The optimal printing orientation for the most accurate measurements and post-processing was found to be 45°. During the rotation of the vessel, the contact points of the support structure were found to have a significant influence on the results. Therefore, it was recommended that an optical sensor should not be placed on or opposite these points.

Although the geometry is relatively simple, the results can be used as a foundation for further research. For example, the knowledge of the most suitable orientation for optical measurements in the 3D printer can be applied to more complex structures, enabling optimal alignment for specific applications.

## Data Availability

No datasets were generated or analysed during the current study.
